# Prof. Warren’s Valedictory Remarks

**Published:** 1847-06

**Authors:** 


*Prof. Warren's Valedictory Remarks.*—At the close of the session of the Medical College in Boston, as we learn by our contemporary, the Boston Med. and Surg. Journal, Dr. Warren "finished his valedictory remarks, by advising medical students not to seek foreign schools, until they should have acquired all the results of deliberate attention at home, which would fit them to profit by the advantages peculiar to other countries. He thought that most students acquired more instruction at home than abroad, especially in countries where a foreign language is spoken. He spoke of the difflculties of obtaining subjects for anatomical inquiry, and proposed that persons, who, like himself, do not object, but prefer to submit their bodies, after death, to anatomical purposes, should, at once, execute a written direction therefor."

"Dr. W. has performed about 5,000 surgical operations, not including minor ones, and delivered 3000 lectures in Boston, and 1000 in Cambridge."

